# Aberrant expression of ADAM9 in ovarian cancer and its clinical significance

**DOI:** 10.1002/jcla.23136

**Published:** 2019-12-03

**Authors:** Ting Guo, Donglan Yuan, Mei Lin, Dandan Zhu, Ning Xu, Jun Wang

**Affiliations:** ^1^ Institute of Clinical Medicine Taizhou People's Hospital Affiliated to Nantong University Taizhou China; ^2^ Department of Obstetrics and Gynecology Taizhou People's Hospital Affiliated to Nantong University Taizhou China; ^3^ Department of Clinical Laboratory Taizhou People's Hospital Affiliated to Nantong University Taizhou China; ^4^ Department of Gastrointestinal Surgery Taizhou People's Hospital Affiliated to Nantong University Taizhou China; ^5^ Department of Emergency Taizhou People's Hospital Affiliated to Nantong University Taizhou China

**Keywords:** A disintegrin and metalloproteinase 9, diagnosis, ovarian cancer, prognosis

## Abstract

**Background:**

The oncogene a disintegrin and metalloproteinase 9 (ADAM9) was up‐regulated in ovarian cancer tissues, and the present study aims to explore the potential diagnostic and prognostic value of ADAM9 in ovarian cancer (OC).

**Methods:**

A total of 30 paired fresh OC tumor tissues and the paired‐adjacent normal tissue, and 90 formalin‐fixed paraffin‐embedded (FFPE) OC samples and adjacent normal tissue were collected. The expression of OC in FFPE samples was examined by immunohistochemical methods, and the mRNA expression of ADAM9 in fresh tumor samples was examined by RT‐qPCR methods. Receiver operating characteristics curve was drawn to analyze the potential diagnostic value of ADAM9. Kaplan‐Meier survival analysis was performed to compare the overall survival (OS) and disease‐free survival (DFS) of the ADAM9 positive and negative OC patients.

**Results:**

The positive rate of ADAM9 in FFPE OC tumor tissue was markedly higher than in the non‐tumorous tissue (61/90 vs 47/90), and increased expression level of ADAM9 may associate with higher histological grade, advanced Figo stage and increased risk of metastasis; moreover, the mRNA expression of ADAM9 was also increased in OC tissue compared with the normal tissue (*P* < .001), and results of ROC analysis suggested that ADAM9 is a sensitive marker for the diagnosis of OC( AUC 0.8389, 95% confidence interval 0.7333 to 0.9445); finally, increased expression of ADAM9 may indicate decreased OS (*P* = .004) and DFS (*P* = .014) of the patients.

**Conclusion:**

A disintegrin and metalloproteinase 9 was up‐regulated in OC, and ADAM9 may serve as potential diagnostic and prognostic marker for the diagnosis and treatment of OC.

## 1 INTRODUCTION

Ovarian cancer (OC) is one of the mostly diagnosed gynecologic cancer worldwide.^1^, ^2^ In current clinical works, like many other type of cancers, OC lack early symptoms, and most of the cases were found in the advanced stages when the patients first came to the hospital.^3^, ^4^ As a result, the survival rate of for women with OC remains low. Based on previous reports, if OC was found at early stage, the survival rate of can reach to 70%‐90%,^3^, ^4^ thus, to search for early diagnostic and prognostic biomarker is of great important to improve the therapeutic efficacy and survival rate of women with OC.

A disintegrin and metalloproteinase (ADAM) proteins are a group of transmembrane proteins that contain a metalloproteinase domain^5^, ^6^ that responsible for releasing cell the cell surface proteins, for examples, the growth factors, cytokines, or the receptors.^5^, ^6^, ^7^ Previous studies suggested that the abnormal expressions of ADAM family proteins were closely correlated with the occurrence and development of different types of cancers.^5^, ^8^, ^9^, ^10^, ^11^ However, studies on the roles of ADAM proteins in OC were limited, and the functions of ADAM proteins in OC still require to be further explored.

A disintegrin and metalloproteinase 9 is a member of the ADAM families, and the roles of ADAM9 in different types of cancers have been discussed previously.^12^, ^13^, ^14^ In the case of OC, ADAM9 was found to be up‐regulated in tumor tissue than the normal tissue^15^; however, the underlying mechanism is still unclear. In the present study, we will examine expression of ADAM9 in human OC tissue by immunohistochemistry and RT‐qPCR method and investigate potential diagnostic and prognostic value of ADAM9 for OC.

## 2 MATERIAL AND METHODS

### 2.1 Patients

The present study included 30 OC tumor tissues and the paired‐adjacent normal tissue that collected between February 2018 and February 2019, and 90 formalin‐fixed paraffin‐embedded OC samples and paired‐adjacent normal tissue that collected between January 2014 and April 2019 from patients who were diagnosed as OC in Taizhou People's Hospital. Patients who have received chemo or radiotherapy before surgery were excluded from this study. The present study has been approved by the ethics committee of Taizhou People's Hospital, and each patient has signed the informed consent form.

### 2.2 Real‐time quantitative PCR

The total RNAs were isolated from the fresh OC tissue and adjacent tissue samples by TRIzol (Invitrogen), and the real‐time quantitative PCR (RT‐qPCR) was performed by the TB Green RT‐PCR kit (TaKaRa). The PCR reaction was performed by an ABI 7500 Real‐Time PCR System (Applied Biosystems), and the condition for PCR was as follows: 95°C, 30 seconds; 40 cycles of 95°C, 5 seconds and 60°C, 30 seconds. The primers were purchased from Sangon Biotech. The relative expression level of ADAM9 was normalized to the expression level of GAPDH by 2^−ΔΔCt^ method. The sequences of the primers were as follows: ADAM9 forward, 5′‐GTGTCCGGTGGTTGCTGT‐3′, ADAM9 reverse, 5′‐AATAGGGCCTAGGGGCTTCTC‐3′; GAPDH forward, 5′‐CTCTGCTCCTCCTGTTCGAC‐3′, GAPDH reverse 5′‐GCGCCCAATACGACCAAATC‐3′.

### 2.3 Immunohistochemical analysis

The OC tumor tissue and the adjacent normal tissue were embedded with paraffin and then sectioned into 4‐mm slides, and the immunohistochemical analysis was performed by the Ready‐to‐Use Immunohistochemistry Hypersensitivity UltraSensitive™ S‐P kit (Maxim). Briefly, the slides were deparaffinized and rehydrated, heat‐fixed with the protein‐blocking solution and then incubated with the primary antibodies (anti‐ADAM9, Boster), and then treated with the HRP‐labeled secondary antibodies. Finally, the tissue samples were stained with the diaminobenzidine (DAB) for colorization and imaged by a microscope.

### 2.4 Histological scoring

The score of the IHC samples was determined by two experienced doctors independently. For the histological scoring, intensity of the staining was ranged between 0 and 3, with negative 0, weak 1, moderate 2, and strong 3. And the positive area of the samples was classified into 0 (positive area <10%), 1 (positive area 10%‐25%), 2 (positive area 25%‐50%), and 3 (positive area >50%). Then, the scoring of each sample was calculated by multiplying the intensity of the staining with the positive area, and 0‐3 represents negative staining and 4‐9 represents positive staining.

### 2.5 Statistical analysis

All statistical analysis was performed by SPSS 17.0 software. Data were presented as the means ± standard deviation, and the paired Student *t* test was performed to for comparison between two groups. Correlation between the expression of the ADAM9 and the clinical characteristic of the OC patients was analyzed by chi‐square test. Receiver operating characteristics (ROC) curve was drawn to evaluate the potential diagnostic value of ADAM9. Kaplan‐Meier survival analysis was performed to compare the overall survival (OS) and disease‐free survival (DFS) of the OC patients in the different groups. *P* < .05 indicated statistically significant.

## 3 RESULTS

### 3.1 Increased expression of ADAM9 in paraffin‐embedded (FFPE) OC tumor samples

**Figure 1:**
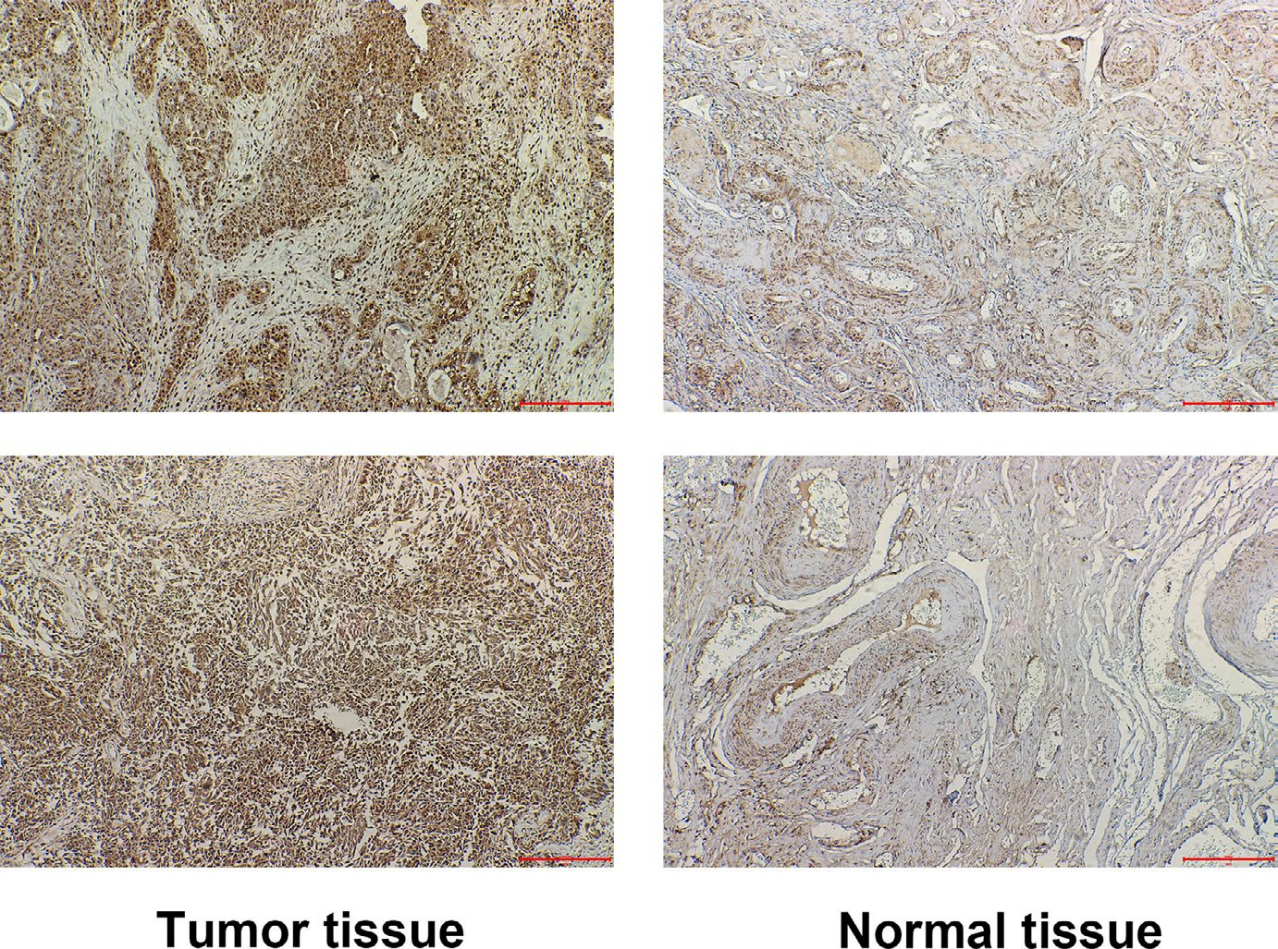
Comparison of the expression of a disintegrin and metalloproteinase 9 in Ovarian cancer tumor and the adjacent non‐tumorous tissue by IHC methods. Scale bar, 200 µmol/L, magnification 100×

First, to explore the roles of ADAM9 in OC, the expressions of ADAM9 in the FFPE OC tumor samples and the adjacent non‐tumorous tissues were compared by IHC methods. As shown in Figure 1 and Table 1, the positive rate of ADAM9 in OC tumor tissue was markedly higher than that in the non‐tumorous adjacent tissue (61/90 vs 47/90, *P* < .05), and increased expression level of ADAM9 was positively associated with the histological grade (*P* < .05), Figo stage (*P* < .05), and metastasis (*P* < .01) of the patients (Table 2).

**Table 1:** Results of the negative and positive numbers of ADAM9 in PFFE tissue samples by IHC methods

	ADAM9		*P* value
	Negative (n)	Positive (n)	
Tumor tissue	29	61	.033*
Non‐tumorous tissue	43	47	

* *P* < .05.

**Table 2:** Clinical characteristics of the patients

Clinical characteristics	ADAM9		*P* values
	Negative (n)	Positive (n)	
Age (years)			
<50	8	15	.761
>50	21	46	
Histological grade			
Well‐modulate	15	45	.038*
Poor	14	16	
FIGO stage			
I/II	16	47	.034*
III/IV	13	14	
Metastasis			
No	10	42	.002**
Yes	19	19	

* *P* < .05.

** *P* < .01.

### 3.2 Increased expression of ADAM9 in fresh OC tumor tissue samples and its potential diagnostic value

**Figure 2:**
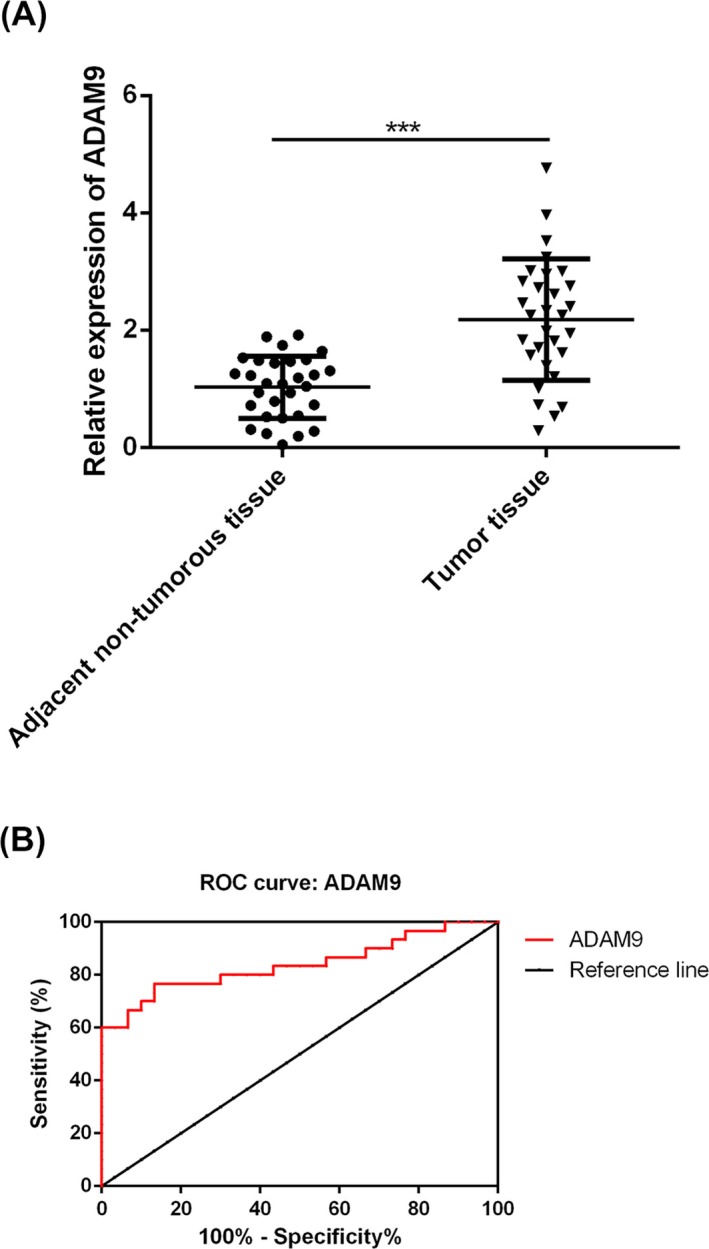
A disintegrin and metalloproteinase 9 (ADAM9) may serve as a diagnostic marker for ovarian cancer (OC). A, Comparison of the mRNA expression of ADAM9 between the OC tumor and the adjacent non‐tumorous tissue by RT‐qPCR methods. B, Results of ROC analysis. ***P < .001

Furthermore, the expressions of ADAM9 in 30 fresh OC tumor samples and the adjacent normal tissue were compared by RT‐qPCR methods. We observed that the expression of ADAM9 was significantly increased in OC tissue compared with the normal tissue (Figure 2A, *P* < .001); moreover, results of ROC analysis suggested that the AUC of ADAM9 for OC was 0.8389 (Figure 2B, 95% confidence interval (CI) 0.7333 to 0.9445), indicating that ADAM9 is a sensitive marker for the diagnosis of OC.

### 3.3 Over‐expression of ADAM9 may indicate poor prognosis of patients with OC

**Figure 3:**
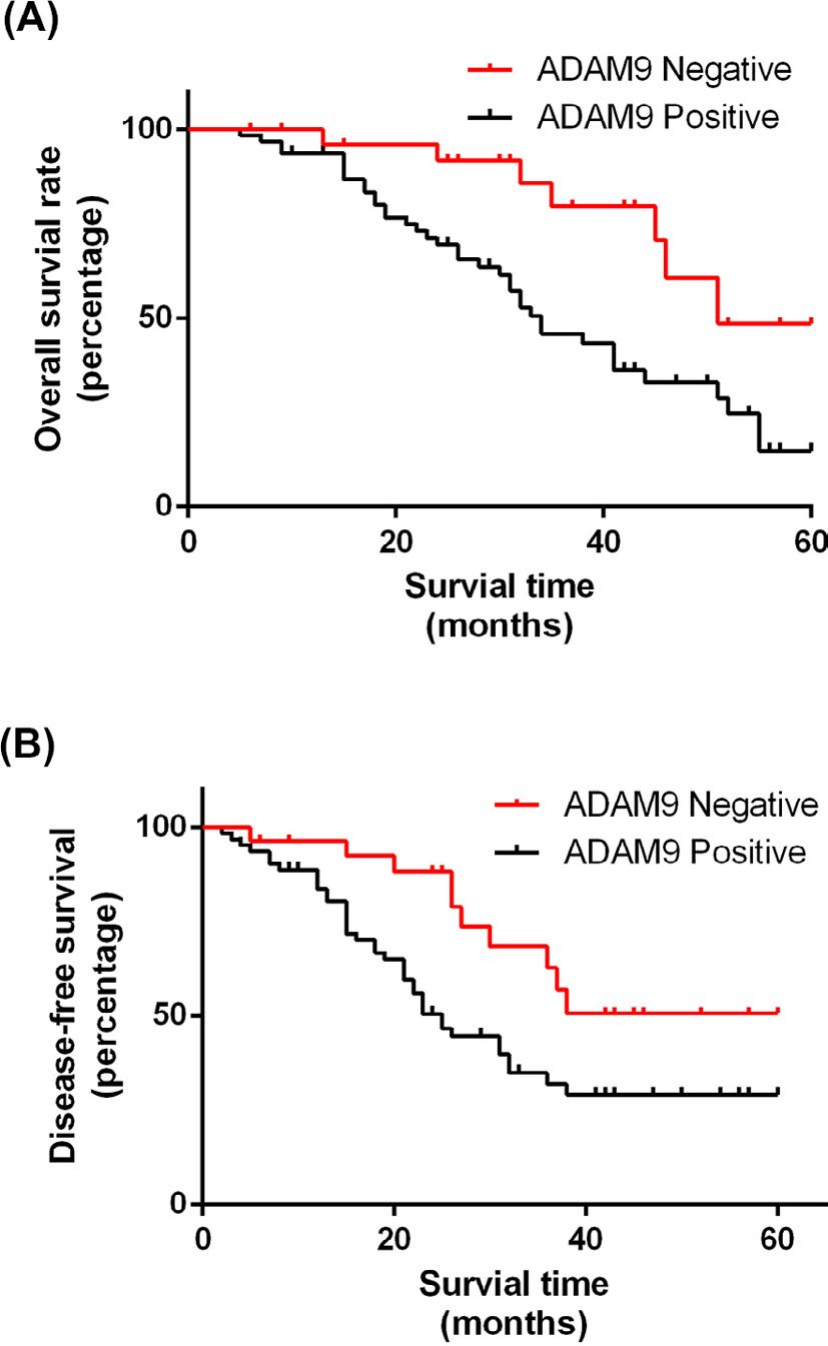
A disintegrin and metalloproteinase 9 (ADAM9) may serve as a prognostic marker for ovarian cancer (OC). A, Comparison of the OS of the ADAM9 positive and ADAM9 negative OC patients. B, Comparison of the DFS of the ADAM9 positive and ADAM9 negative OC patients

Finally, we performed Kaplan‐Meier survival analysis to investigate the roles of ADAM9 expression in the evaluation of OS and DFS of the OC patients. As shown in Figure 3, both the OS (Figure 3A, *P* = .004) and the DFS (Figure 3B, *P* = .014) of OC patients with significantly lower in the ADAM9 positive group compared with the ADAM9 negative group, which suggested that increased ADAM9 may indicate poor prognosis of OC patients.

## 4 DISCUSSION

In the present study, we have explored the roles of ADAM9 in OC and its clinical significance. We found that ADAM9 was significantly up‐regulated in OC tissue compared with the normal tissue, and we also demonstrated that AMAM9 may serve as potential diagnostic and prognostic marker for the early diagnosis and treatment of OC.

The roles of ADAM9 in different types of cancers have been discussed in many previous works. Caporali et al suggested that microRNA‐126‐3p may contribute to the dabrafenib resistance of melanoma via up‐regulating ADAM9 Caporali^16^; Oria et al found that ADAM9 may contribute to the development of pancreatic ductal adenocarcinoma^17^; Dong et al demonstrated that ADAM9 can induce the epithelial‐mesenchymal transition of the hepatoma cells^18^; Wang et al suggested that ADAM9 functions as an oncogene in gastric cancer, and it was negatively regulated by micoRNA‐126.^14^ A study on the roles of ADAM9 in OC is limited. Ueno et al reported that ADAM9 is over‐expressed in OC, and may contribute to the cisplatin sensitivity of OC cells.^15^ In the present study, we observed that the positive rate of ADAM9 in PFFE OC tissue was significantly increased in tumor tissue compared with the adjacent tissue, which was consistent with the Ueno et al's observation; moreover, the expression of ADAM9 was positively associated with a higher histological grade, Figo stage, and metathesis of the tumor. Taken together, these data indicated that ADAM9 was up‐regulated in OC and may function as an oncogene.

The early diagnosis of OC is of great importance to increase the survival of the patients.^19^, ^20^, ^21^ It is still unclear whether ADAM9 can serve as potential diagnostic biomarker for OC. In this study, we further explored the potential diagnostic value of ADAM9 using fresh OC tissues. We found the expression level of ADAM9 was markedly decreased in the OC tissue samples compared with the normal controls, and ROC analysis confirmed that ADAM9 can distinguish the OC tumor tissue from the adjacent non‐tumorous tissue. These results suggested that ADAM9 may serve as potential diagnostic marker for OC.

Previous studies also suggested that ADAM9 may function as prognostic marker in different diseases. For example, Fan et al found that ADAM9 may act as a prognostic marker in lower‐grade gliomas^22^; Kossmann et al suggested that the high expression level of ADAM9 may indicate poor prognosis in lung adenocarcinoma.^23^ However, the potential roles of ADAM9 in OC have not yet been discussed. In the present study, we found that both the OS and the DFS of OC patients with significantly lower in the ADAM9 positive group compared with the ADAM9 negative group. These results suggested that ADAM9 may serve as a prognostic marker for OC, which may be beneficial for the treatment of the disease.

Our study has limitations. First, because of ethical issues, we only collected 30 fresh OC tissue samples, and the results should be investigated with larger samples size in future studies; second, the underlying mechanism of ADAM9 in OC as an oncogene should be further explored by cellular and animal studies.

In summary, we found that ADAM9 was up‐regulated in OC, and we first reported that ADAM9 may serve as potential diagnostic and prognostic marker for OC. Our data have provided new evidence for the potential application of examining the expression level of ADAM9 for the early diagnosis and treatment OC.
